# The Huntington procedure: still a reasonable option for large tibial defects in paediatric patients

**DOI:** 10.1007/s11832-014-0618-8

**Published:** 2014-10-29

**Authors:** Som P. Gupta, Gaurav Garg

**Affiliations:** 1 Mahatma Gandhi Medical College and Hospital, RIICO Institutional Area, Sitapura, Jaipur, 302022 Rajasthan India; 2 S.M.S. Medical College and Attached Hospital, Jaipur, 302004 Rajasthan India

**Keywords:** Huntington, Defect non-union, Tibialization, Fibula, Paediatrics

## Abstract

**Background:**

Management of gap nonunion of tibia is technically difficult, time consuming, physically and psychologically demanding for the patient with unpredictable results. Various techniques have been described in literature for the treatment of gap nonunions, but each one has its own limitations.

**Purpose:**

This study reports the outcomes of ipsilateral fibular transposition for reconstruction of tibial defects in paediatric age group.

**Methods:**

We retrospectively reviewed records of 14 patients who underwent surgery for gap nonunion tibia with ipsilateral tibialization of fibula. Fibula is transferred to tibia as pedicle graft in two-staged procedure. Due to retained blood supply to one end of the transplant, the graft easily takes up and hypertrophies upon weight bearing over a period of time.

**Results:**

Average time of radiographic union was 13.35 weeks. Guarded partial weight bearing was started at an average of 16.5 weeks with gradual progression to full weight bearing. The leg length discrepancy at final follow-up ranged from 0 to 7 cms with an average of 2.60 cms. Hypertrophy of tibialized fibula was observed in all patients, with 8 patients showed grafted fibula reaching the diameter of opposite tibia. On subjective assessment, 9 patients were highly satisfied, 4 patients were satisfied and one patient was dissatisfied with the procedure.

**Conclusions:**

Huntington procedure is a simple, cost-effective and easy procedure for large tibial defects in paediatric patients which does not require any specialized team and implants, and can be performed in moderately equipped hospital. Leg length discrepancy may be addressed, after the transferred fibula is well developed.

## Introduction

Children with segmental defects of tibia may present with a wide range of clinical entities, and the defect may also be associated with old or active osteomyelitis, compromised vascularity, scarring due to previous intervention, soft tissue injury and malalignment of the limb, among others. The clinical management of gap nonunion of tibia in association with these manifestations is not only difficult in terms of planning the treatment regimen, but it is also time-consuming, physically and psychologically demanding for the patient and associated with unpredictable results. In the past, cases with wide bone loss following severe injuries or infections often required amputation; today, such bone trauma can be salvaged by surgical techniques, such as segmental bone transport, auto graft or allograft transplantation, the Ilizarov frame and microvascular surgery [1]. However, because each of these techniques is characterized by its own unique limitations which may compromise the final functional results, new approaches are constantly being sought. In spite of multiple attempts to develop new procedures, for patients with persistent nonunion, the risk of infection may increase and can lead to an amputation of the leg.

Transposition of the ispilateral fibula to the tibia (fibula pro tibia) was suggested by Hahn [2] in 1884 and was first used successfully by Huntington [3] in 1903 to fill a 12.7-cm tibial defect in a 7-year-old boy. Subsequently, several authors have used similar techniques [4–6]. The technique which we describe here involves the transfer of fibula to tibia as a pedicle graft in a two-stage procedure. Due to the retention of the blood supply to one end of the transplant, the graft easily takes up and hypertrophies upon weight bearing over a period of time [3, 4]. This procedure was chosen for study as it does not require long-term treatment nor external fixator care associated with segment transport. This method is usually recommended for paediatric patients as the fibular graft in adult patients is considered to be inadequate in terms of size and strength and is therefore liable to fracture [8].

The aim of this study was to report the outcomes of ipsilateral fibular transposition using the Huntington procedure for the reconstruction of tibial defects in paediatric patients.

## Methods

This was a retrospective study in which the medical records of 14 paediatric patients who had undergone surgery for gap nonunion of tibia with ipsilateral tibialization of the fibula between 1998 and 2003 were reviewed. The radiographs, clinical records and functional status of these 14 patients (10 boys, 4 girls), who ranged in age from 2 to 13 (average 8.35) years, were evaluated. All of these patients had tibial defects, either secondary to massive diaphyseal sequestration after osteomyelitis (infection group; 10 patients) or following open fracture of the tibia (trauma group; 4 patients). One patient in the trauma group had an ipsilateral open femur fracture with degloving injury to the popliteal region. One patient in the infection group had proximal tibiofibular joint dislocation, and another had pre-operative varus deformity at the ankle joint. All patients had segmental tibial loss ranging from 4.5 to 11 (average 7) cm. The inclusion criteria were patients aged <15 years with a defect of >4 cm, an intact fibula, no distal neurovascular deficit, no contraindication for surgery, an adequate tibial stump at both the proximal and distal level for fixation with fibula and a minimum follow-up period of 5 years. Two patients with similar clinical criteria were treated primarily with Ilizarov’s technique: one was lost to follow-up during the treatment and even did not turn up for frame removal, and the other patient, although doing well, was admitted to hospital five times due to problems related to frame readjustment and pin site infection.

The details of the initial treatment method and the number and type of operations performed prior to the tibialization of fibula were recorded. Patients were subjected to routine investigations for surgery, and X-rays were obtained of the affected leg. A segmental bony defect was defined when there was no radiographic evidence of contact between the bone ends on either side of the nonunion on two orthogonal views. If a gap was present, it was measured from the point at which at least 50 % of the diameter of the bone was present in the proximal fragment to the corresponding point on the distal fragment on a radiograph. In patients with chronic osteomyelitis, initially active infection was managed with serial debridements followed by intravenous antibiotics. Before starting treatment, the patient’s guardians were made to understand the real situation as well as possible future outcomes. Informed consent was obtained from all families.

**Fig. 1:**
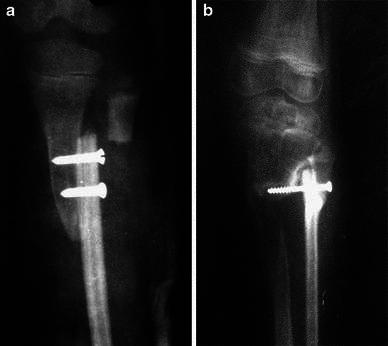
**a** Radiograph of an 8-year-old child showing proximal fixation of the fibular graft using two screws. **b** Radiograph of a 9-year-old child showing proximal fixation of the fibular graft using one screw

The first stage of our surgical procedure includes synostosis between the proximal tibial fragment and the fibula. Two incisions are used—a lateral incision to expose the proximal fibula and an anterior incision to expose the distal lateral part of the proximal tibia. The area is chosen based on the lateral aspect of the tibia, which should be free of scarring and infection, and a trough is prepared to receive the proximal end of fibula by slanting the small osteotome upwards and inwards. The fibula is osteotomized at a slightly higher level than proximal edge of the tibial trough. The proximal end of the fibular graft is fitted into the trough and secured with one or two screws (*n* = 5) or k-wires (*n* = 6) (Fig. 1a, b). In three of our patients, both k-wires and screws were used for fixation. As we proceed subperiosteally at the osteotomy site and the remaining muscular attachments to the fibula are intact, the fibula is not devascularized completely and there is always a free blood supply to the other end of the transported fibula.

Following surgery, the leg is externally immobilized a long leg cast for 6–8 weeks, by which time union is generally evident on the radiography images (radiographic union), primarily based on the presence of uninterrupted external bridging callus at the syostosis site with no gap in between. The second stage of the procedure is conducted in a similar fashion as the first by fixing the distal end of the fibular graft into the prepared slot and reinforcement with screws or k-wires. Immobilization in the long leg cast continues until radiographic union is evident, usually by 10 weeks post-surgery. Guarded partial weight bearing starts with the patient using the polypropylene patellar tendon-bearing top orthosis until sufficient hypertrophy of fibula is evident on radiographs.

Our patients were followed every 3 months for 1 year and every 6 months thereafter. They were assessed clinically, and radiographs of the affected tibia were taken at each visit. Results at the last follow-up were analyzed based on functional, clinical and radiological outcomes. Patients and their guardians were asked to subjectively assess their level of satisfaction with the final result as highly satisfied, satisfied or dissatisfied. The assessments were based on school attendance, outdoor playing, daily routine activities (i.e. squatting, climbing stairs, doing strenuous work), and pain score [using a 10-point visual analog scale (VAS) where 1 represents no pain and 10 represents unbearable pain]. The range of motion of the knee and ankle at the final follow-up, clinical alignment of the knee and ankle joint and final shortening were also taken into account.

## Results

**Fig. 2:**
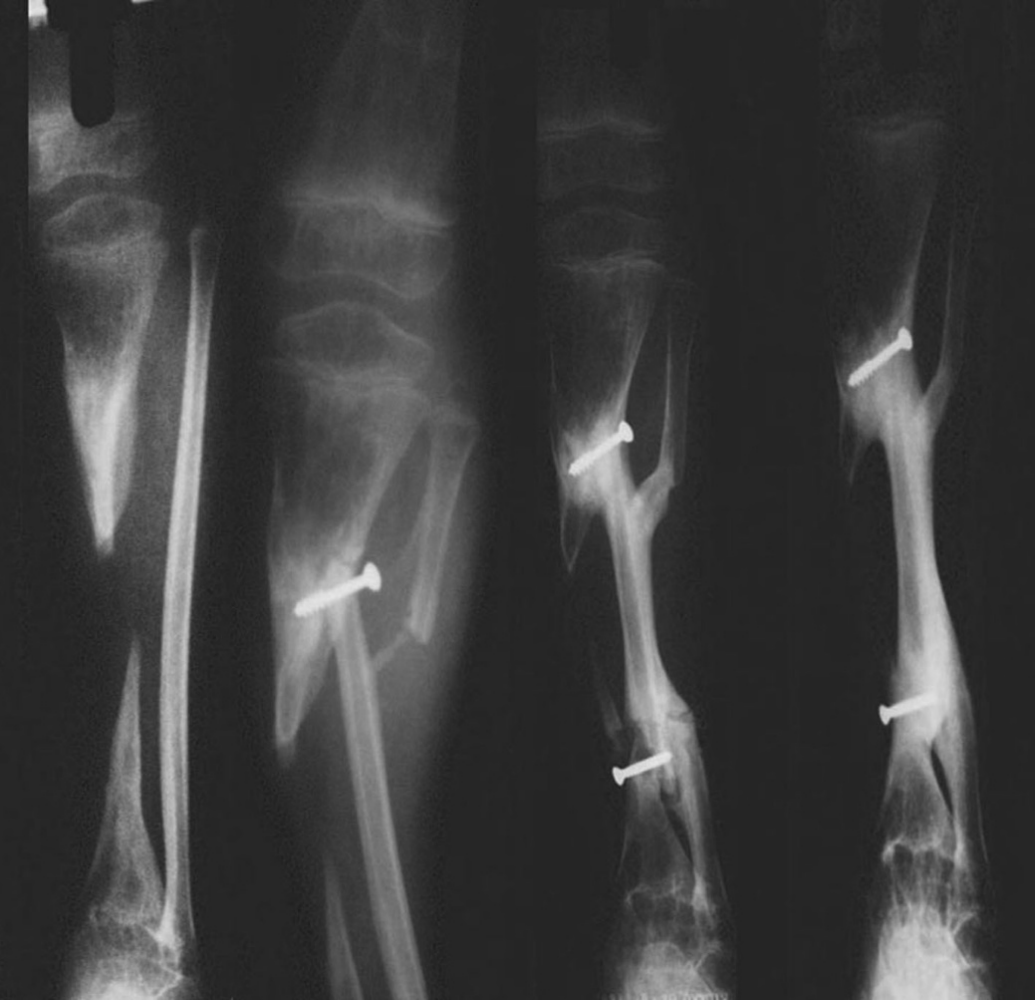
Radiographs of 10-year-old boy showing progressive consolidation of fibular graft and hypertrophy of tibialized fibula 4 years post-surgery

**Fig. 3:**
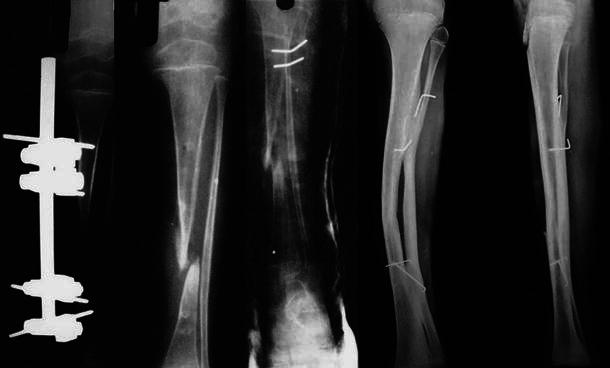
Radiographs of 7-year-old body showing pre-operative X-ray with external fixator in situ, tibialization of fibula and radiographic union at final follow-up at 9 years post-surgery showing good consolidation and hypertrophy of fibula

Two-staged tibilization of fibula was performed In all 14 paediatric patients. The transferred fibula fused well, both proximally and distally, in all patients, without major complications. All patients experienced satisfactory healing of the soft tissue over the tibia, and there were no cases of fracture of the transplanted fibula. Tibial defects occurred due to chronic osteomyelitis (10 patients) and as a consequence of open fracture of tibia (4 patients). The time interval between the first and second stage of the surgical procedure ranged from 4 to 15 (average 6.85) weeks. The average time to union noted radiographically (radiographic union) was 13.35 (range 9–22) weeks (Table 1). Guarded partial weight bearing was started at an average of 16.5 (range 11–25) weeks post-surgery, with gradual progression to full weight bearing. The follow-up period after the second stage of fibular transposition ranged from 6 to 15 (average 9.71) years (Table 1). The leg length discrepancy at the final follow-up ranged from 0 to 7 (average of 2.60) cm. Hypertrophy of tibialized fibula was observed on the radiographs of all patients, with eight patients showing grafted fibula reaching the diameter of the opposite tibia (Figs. 2, 3).

**Table 1:** Patient characteristics, complications and results

Subject no.	Age (years)/sex	Length of tibial defect (cm)	Length of time to achieve radiographic union (weeks)	Length of time post-surgery at initiation of weight bearing (weeks)	Total follow up (years)	Complications	Length of leg shortening at final follow-up (cm)	Results of patient satisfaction
1	11/Male	11	18	20	8	Breakage of proximal k-wire, 20° lateral and 35° posterior angulation, Lateral subluxation of knee	5	S
2	8/Female	9.5	11	14	13	-	3	HS
3	2/Male	6.5	9	11	6	-	2	HS
4	7/Male	5.5	15	15	9	-	0	HS
5	10/Male	10	22	25	8	30° Proximal tibial varus, proximal fibular migration	5	DS
6	13/Female	5	10	12	14	-	3	HS
7	9/Male	6.5	12	14	9	Proximal screw cut through	1.5	HS
8	10/Male	6.5	21	25	11	-	2	HS
9	8/Male	7	14	20	15	30° varus at ankle	4.5	S
10	7/Female	7.5	10	14	7	-	3.5	HS
11	9/Male	5	15	21	10	-	0	HS
12	12/Female	4.5	11	13	9	-	2.5	HS
13	4/Male	7	9	12	11	Recurrence of infection	4	S
14	7/Male	6.5	10	15	6	-	1.5	S

*HS* Highly satisfied, *S* satisfied, *DS* dissatisfied

**Fig. 4:**
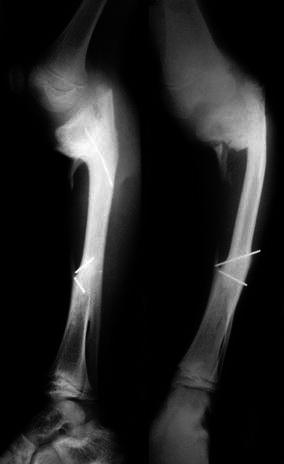
Lateral and antero-posterior radiographs of a 10-year-old boy showing marked varus and posterior angulation at the knee with proximal migration of the fibula, leading to shortening and stiffness of both knee and ankle joints

**Fig. 5:**
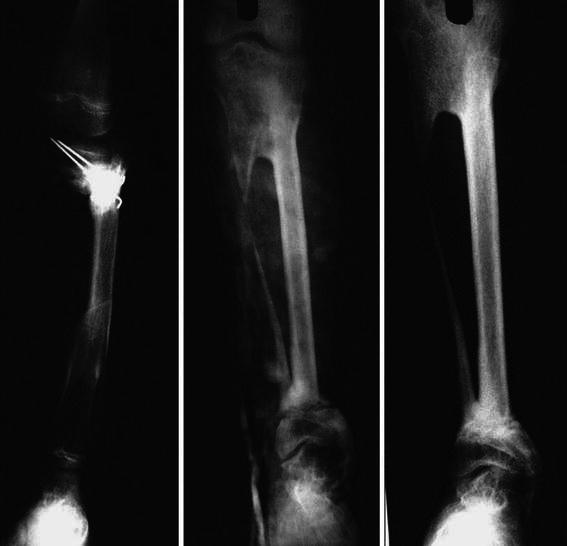
Radiograph showing additional procedures for the correction of the proximal tibial varus deformity at the knee and marked varus deformity at the ankle joint in two different patients

Results at the last follow-up were analysed based on functional, clinical and radiological outcomes (Table 2). Patients were asked at the last follow-up to subjectively assess the results. Nine patients were highly satisfied, four patients were satisfied and one patient was dissatisfied with the procedure due to marked varus at the knee following damage to the proximal tibial epiphysis and uninterrupted growth of the proximal fibular epiphysis, leg shortening of 5 cm and stiffness of both knee and ankle joints (Fig. 4). When the defect was very near to either end of tibia, it was difficult to fix the fibular graft to the short tibial segment. The proximal fragment was very small in two patients, and the k-wire was inserted with difficulty. There was breakage of the proximal k-wire in one patient, and the proximal screw had to be cut out in another patient, but fortunately the synostosis site healed well. Other complications encountered were recurrence of infection, varus angulation at the ankle and lateral subluxation of the knee. Two patients required additional surgeries—one for correction of proximal varus deformity and another for correction of marked varus at the ankle joint following hypertrophy of the fibula (Fig. 5). Recurrence of infection in one patient was treated with intravenous antibiotics. At last follow-up for documentation, four patients had undergone an additional procedure for limb equalization by the Ilizarov technique. However, upon retrospectively reviewing our results, we observed that among our patients tibial gaps of >6.5 cm were associated with less favorable outcomes than those <6.5 cm.

**Table 2:** Functional and clinical outcomes for each patient

Subject no.	Attendance at school	Outdoor playing	Daily activities^a^			Pain score^b^	Knee movements		Ankle movements		Knee alignment^c^	Ankle alignment^c^	Results of subjective assessment
			Squatting	Climbing stairs	Strenuous work		Extension	Flexion	Dorsiflexion	Plantarflexion			
1	Yes	Yes	b	a	a	2	10°	85°	20°	30°	10° varus	15° valgus	S
2	Yes	Yes	a	a	a	1	0	120°	30°	40°	n	n	HS
3	Yes	Yes	b	a	a	1	0	90°	20°	20°	n	n	HS
4	Yes	Yes	a	a	a	2	0	100°	10°	40°	n	n	HS
5	Yes	No	c	a	c	6	15°	40°	0	30°	30° varus	n	DS
6	Yes	Yes	a	a	a	1	0	110°	20°	50°	n	n	HS
7	Yes	Yes	a	a	a	1	0	110°	10°	50°	n	n	HS
8	Yes	Yes	a	a	a	1	0	100°	20°	20°	n	n	HS
9	Yes	Yes	b	b	b	8	10° extension lag	90°	10°	10°	n	35º varus	S
10	Yes	Yes	a	a	a	2	0	100°	20°	40°	n	n	HS
11	Yes	Yes	a	a	a	2	0	115°	15°	35°	n	n	HS
12	Yes	Yes	a	a	a	1	0	100°	10°	45°	n	n	HS
13	Yes	Yes	b	a	b	4	10° extension lag	100°	10°	20°	n	n	S
14	Yes	Yes	b	b	c	2	0	80°	5°	20°	n	n	S

^a^'a', patient carried out activity without difficulty; 'b', patient carried out activity with difficulty; 'c', restricted activity

^b^Pain score was assessed using a visual analogue scale where 1 represents no pain) and 10 represents unbearable pain

^c^'n', Normal alignment at knee or ankle joint

## Discussion

The treatment of large segmental defects of the tibia can be challenging to both the treating orthopaedic surgeon and the patient [3, 6, 9, 10]. Such defects are most commonly caused by neglected and chronic osteomyelitis in developing countries, followed by accidents and tumor resection. Various methods of treatment have been used to manage such cases, including the Papineau procedure, allograft reconstruction, distraction osteogenesis, vascularized or nonvascularized contralateral fibular transfer, Huntington’s procedure, among others. Any reliable evaluation of a reconstruction technique should address various factors, including the ease of the procedure and its associated morbidity, complications, functional outcome and durability. Treatment must be based on sound principles of good fixation, control of infection and an environment favourable to bone healing [11]. Large defects of an infectious etiology constitute a major problem, and the management of such cases results in a less favorable outcome compared with that of uninfected defects [12]. The surgical team must therefore ensure complete elimination of the infection in the tibia before reconstructive procedures are commenced.

In lower animals, the fibula is considered to be an important supporting structure in the leg. In contrast, during the course of human evolution and the transition in ambulation pattern from digitigrade to straight plantigrade walking, the human fibula has lost its shine. However, Lambert [13] showed that one-sixth of the static load of the leg is carried by the fibula. When the fibula is subjected to more than normal weight-bearing stresses, it undergoes hypertrophy and become an integral part of the static supporting architecture of the leg. A fibula that is transferred as pedicle graft to bridge gap nonunion in ipsilateral tibia will hypertrophy under stresses and substitute for the defective tibia.

Various other techniques have been described for the treatment of gap nonunions of the tibia, but each one has its own unique limitations. For example, the Papineau type of cancellous bone grafting allows complete excision of infected tissue and rapid vascularization of the graft, but it is associated with poor mechanical strength and a limited amount of graft and requires a long hospital stay with staff experienced in the bone site irrigation technique. Application of the Ilizarov frame provides early weight bearing and good alignment of the limb during the bone transfer phase that reduces disuse osteoporosis and stiffness of the adjacent joints. The options of bifocal or trifocal bone transport with acute or gradual docking to treat defects are also available. However, these procedures are time consuming, add to number of steps with added complication rates and patient compliance is poor, especially in paediatric patients. Babar et al. [14] conducted a study on 32 patients with segmental loss of tibial diaphysis treated by distraction osteogenesis and reported that nine patients developed pin tract infection and seven patients required surgical intervention. The use of bone transport to fill large gaps is reliable but requires the patient to be put on a frame for long periods of time. There are also chances of misdirection during osteogenesis and skin injury, and the gap may not fill at one go. In addition, regular monitoring of bone growth, joint mobility, and any pin tract infection, loosening, or breakage is essential, as is the need for rigorous physiotherapy both during the frame period and after its removal.

Free vascularized fibular grafts from the contralateral limb heal quickly and the chances of infection are less. Also, these grafts survive on their own and do not undergo creeping substitution. On the downside, this procedure is associated with a relatively higher morbidity to the normal limb and requires a specialized microsurgical team which may not be possible in every center. Free vascularized grafts from the opposite leg may necrose in the presence of sepsis, and even if the graft does survive, healing will take a very long time, with expectation of a poor functional outcome.

An avascular strut allograft reconstruction is a well-accepted procedure for gaps created following tumor resections, but this procedure is limited by its general availability, length of resection, risk of nonunion, fracture and infection, as well as by the risk of disease transmission [15, 16]. Also, soft tissue closure can be a problem in this procedure due to the difficulties in closing low-quality tissues over a relatively large construct. Views still clash on providing primary or secondary amputation to curtail the period of suffering.

As early as 1905, Huntington [3] described a technique of fibular transposition and mentioned the advantages of utilizing the fibula as graft. He popularized the technique as a two-stage procedure in 1944 for the treatment of tibial defects in children. A large graft of ipsilateral fibula which is first raised onto the pedicle of the peroneal artery and then aligned and fixed to the tibia in posterior aspect provides a sound mechanical and biological basis for the union [17]. The fibula is completely surrounded by muscles and has an abundant vascular supply which supports its hypertrophy and union at the synostotic site [17, 18]. The reduction in lower leg volume due to the antero-medial shift of the fibula makes skin closure easier, even in patients with scarred tissue [19]. The procedure is restricted to the ipsilateral limb, unlike those cases in which contralateral fibula is used as a vascularized graft, which helps reduce morbidity. Retained vascularity of the graft reduces infection, improves the chances of union and accelerates the process of hypertrophy [20]. In our series, patients underwent fixation with screws or k-wires. Only one patient had a cut-through screw, but the synostosis site healed well, and no intervention was done. Puri et al. suggested that the transposed fibula retains its biological potential but lacks stability [7].

This procedure has been criticized for its two-stage surgery. Also, in the event of nonunion at either site, the splinting action of the fibula will be lost, leaving a failed limb. As an alternative, a synostosis operation has been designed which consists of the one-stage substitution of defective tibia with the fibula, with or without the addition of the sliding or inlay graft. This option may do fairly well in the treatment of adult nonunions, but Huntington’s procedure is still a reasonable option for gap nonunions of tibia in paediatric patients. For a single-stage procedure to be successful, fibula would need extensive release from the lateral surface both proximally and distally, which both jeopardies its blood supply and makes the construct unstable. In the two-stage procedure, even if the extensive soft tissue release is performed only at one synostosis site, the fibula blood supply from other end will remain intact, facilitating healing at the synostotic site. Ozaki et al. [21] concluded that the fibula is unable to withstand the stress of weight bearing. In our series, we did not encounter any stress fracture of the fibula. Guarded weight bearing was started based on radiological union, and hypertrophy developed late after continued weight bearing. Krieg et al. [22] showed that hypertrophy of fibula is common in younger patients and the chances of fatigue fracture are less, which may explain the good fibular support which we observed in all of the patients in our study as all were aged <15 years.

## Conclusion

Our experience shows that Huntington’s procedure still holds a unique place for the treatment of large gap nonunions of tibia, especially in paediatric patients, even though relatively large gaps may be a prognostic factor for poor outcome. Tibialization of fibula is a simple, cost-effective and easy procedure which does not require any specialized team and implants, and it can be performed in a moderately equipped hospital. In our series of patients, fixation was achieved with one or two stainless steel screws or k-wires, which reduces the implant cost and does not require the involvement of microsurgical expertise. A hospital stay of only 1–2 days is required for each synostosis procedure, thus making a total hospital stay of 2–4 days. Frequent monitoring is not required during the follow-up, thus reducing the cost of travelling.

Huntington’s procedure has several advantages: operative dissection of the fibula is minimal, the fibula remains well vascularized as the blood supply is not dissected and muscle attachments are maintained. We believe that for good results regarding the management of tibial nonunions, no single method of treatment should be given preference over any other, rather the entire treatment needs to be individualized by the treating surgeon, with due consideration to soft tissues, socio-economic factors and available expertise. Any discrepancy in leg length may be addressed after the transferred fibula is well developed.
